# Supplementary material for: Conservation of dark CPD photolyase function in blind cavefish
Source: Nat Commun. 2025 Aug 11;16:7377. doi: 10.1038/s41467-025-62795-7 (PMC12340131; doi:10.1038/s41467-025-62795-7)
Supplement: Supplementary file 1 — Supplementary Information [file 41467_2025_62795_MOESM1_ESM.pdf]

Conservation of dark CPD photolyase function in blind cavefish

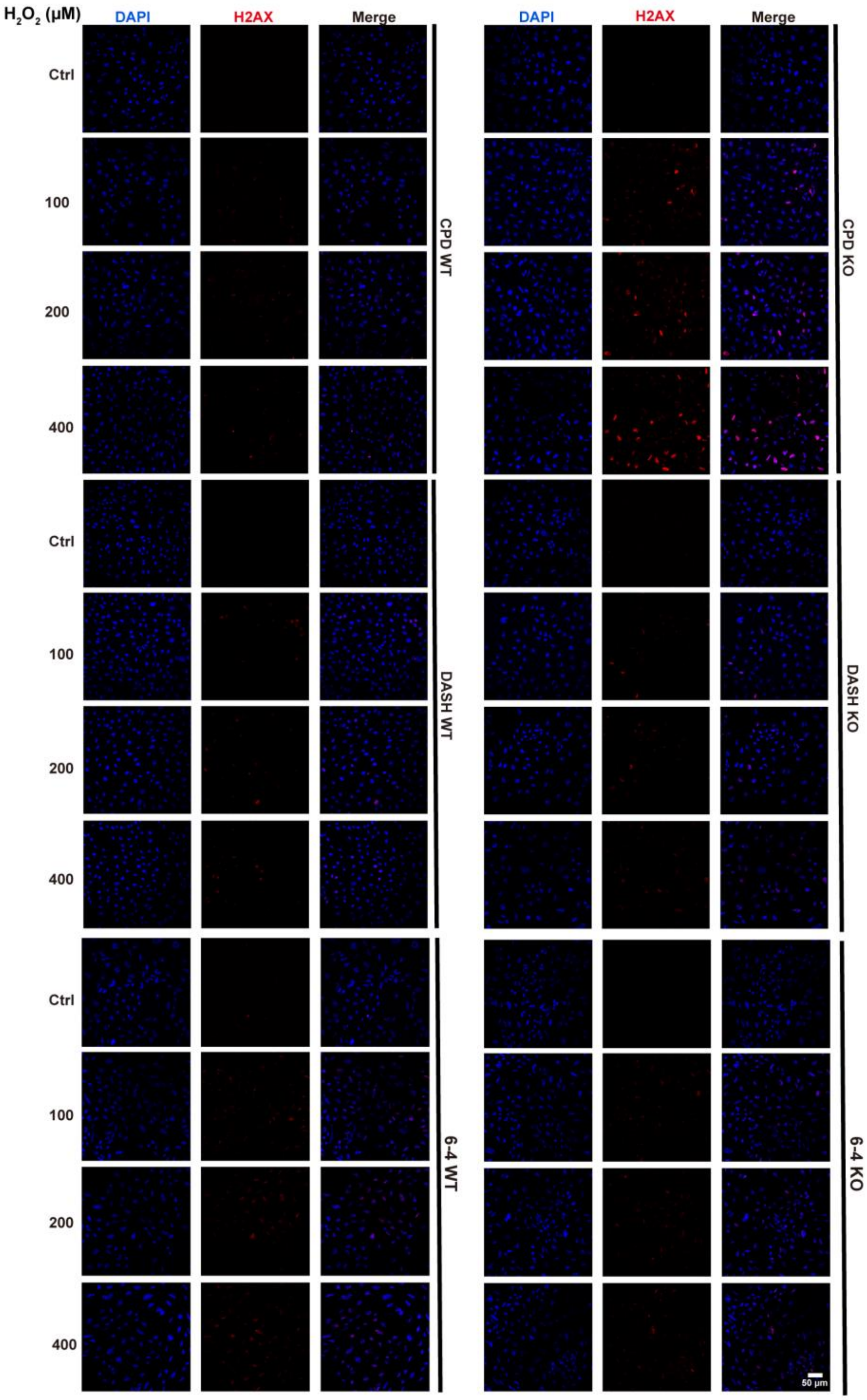

<u>**Supplementary Figure 1**</u> **Loss of CPDphr function in medaka cells results in increased DNA damage upon oxidative stress.** Representative confocal images of medaka photolyase WT and CPDphr, DASHphr and 6-4phr KO cells (CPD KO, DASH KO and 6-4 KO respectively) immunostained for γ-H2AX (Red) and DAPI (Blue) as well as merged images (Merge) following treatment with various concentrations of H_2O_2 (μM) or in non-treated controls (Ctrl) (as indicated on the left-hand side).

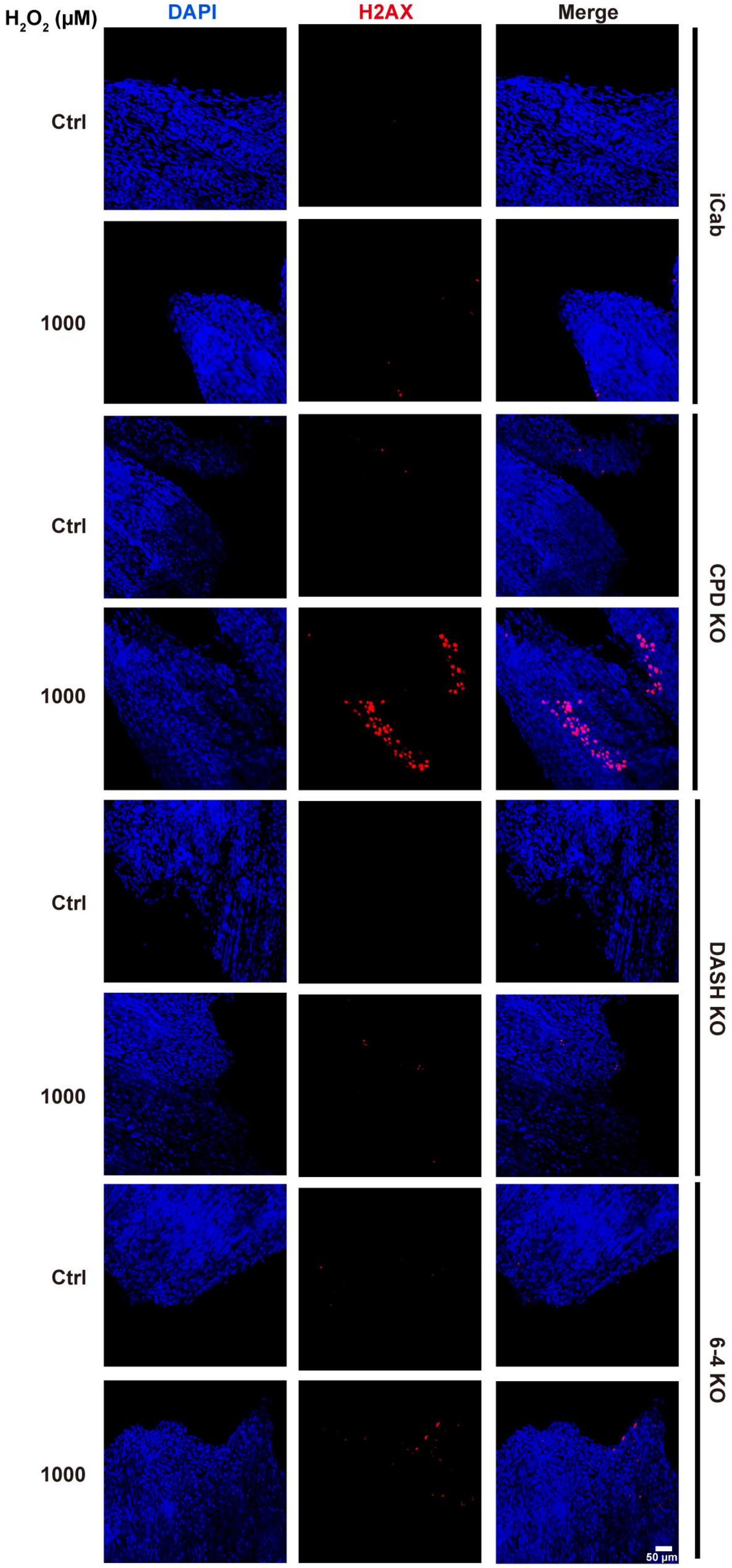

H2O2 (μM)
DAPI
H2AX
Merge
Ctrl
1000
Ctrl
1000
Ctrl
1000
Ctrl
1000
iCab
CPD KO
DASH KO
6-4 KO
50 μm

24

<u>Supplementary Figure 2</u> Loss of CPDphr function in medaka fin clips results in increased DNA damage upon oxidative stress. Representative confocal images of medaka WT and CPDphr, DASHphr and 6-4phr (CPD KO, DASH KO and 6-4 KO respectively) photolyase mutant fish fin clips immunostained for γ-H2AX (Red) and DAPI (Blue) as well as merged images (Merge) following treatment with 1000μM H_2O_2 or in non-treated controls (Ctrl) (as indicated on the left-hand side).

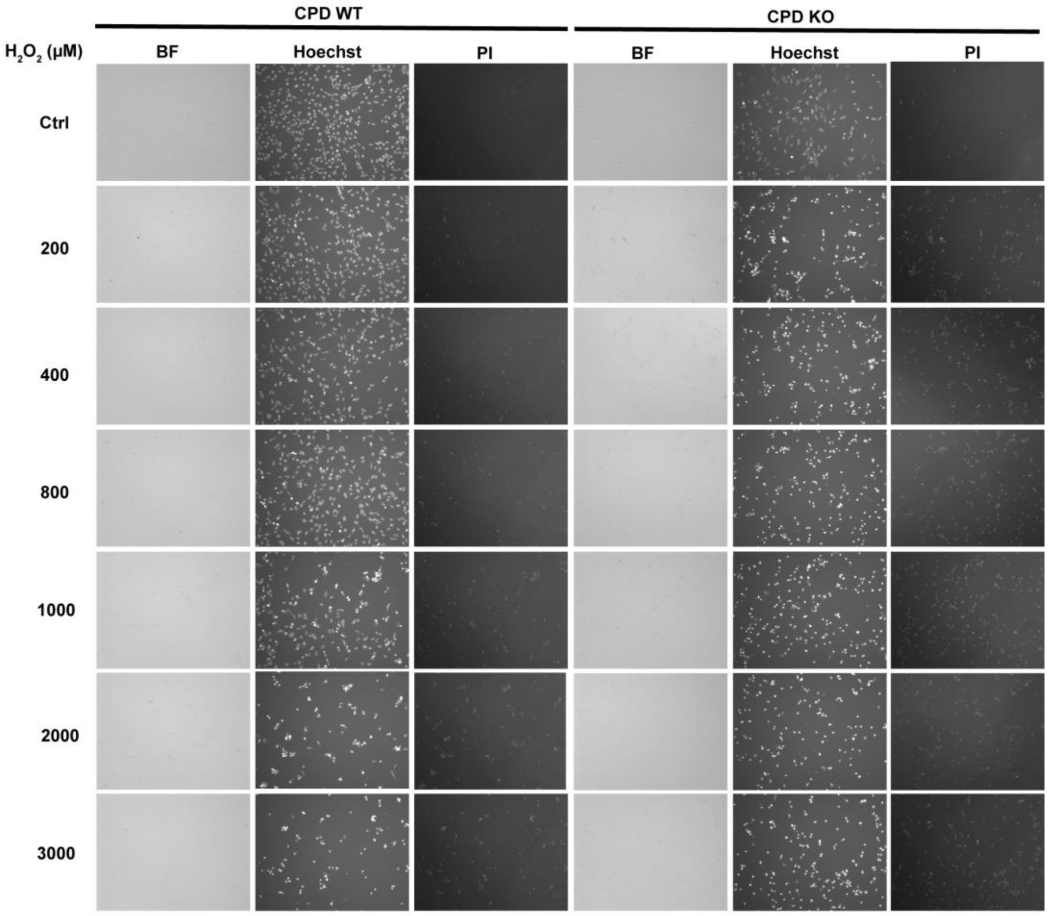

<u>Supplementary Figure 3</u> Loss of CPDphr function in medaka cells results in increased cell mortality upon oxidative stress. Representative images from the automated high-throughput microscopy (AHM) assay of wild type and CPDphr mutant medaka cells (CPD WT and CPD KO respectively) following treatment with various concentrations of H_2O_2 (μM) or in non-treated controls (Ctrl) (as indicated on the left-hand side). Bright field (BF) channel, Hoechst staining, and Propidium Iodide (PI) staining are represented.

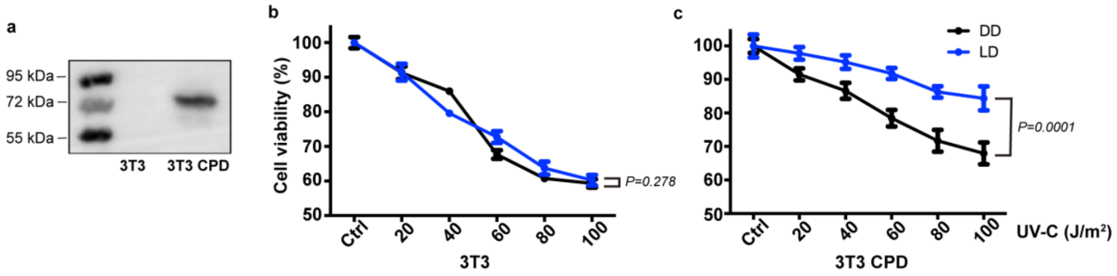

<u>Supplementary Figure 4</u> Gain of CPDphr function in mammalian cells confers enhanced cell survival following UV treatment upon exposure to light. (a) The expression of myc-tagged zebrafish CPDphr in mammalian cells confirmed by western blotting. An expression vector for zebrafish CPDphr was stably transfected into 3T3 cells which were then selected for Neomycin resistance (3T3 CPD). Non-transfected 3T3 cells were employed as a negative control (3T3). **(b-c)** Cell viability assay of 3T3 cells ectopically expressing CPDphr (3T3 CPD) which conferred photoreactivation repair of UV-induced DNA damage. DD indicates cells maintained in constant darkness, while LD represents exposure to a light-dark cycle. Mean percentage ± SEM (n=8 biologically independent samples) of cell viability with respect to untreated cells is plotted on the y-axes, while UV-C light doses (from 20 J/m^2 to 100J/m^2) are indicated on the x-axes. Controls confirming that the transfection and subsequent selection procedures are not responsible for the observed results with the 3T3 cells are provided in Supplementary Figure 10, (b) and (c) where mutant forms of CPDphr were ectopically expressed in 3T3 cells and results equivalent to those from untransfected 3T3 cells were obtained. All experiments were repeated at least 3 times, independently and representative data is shown. The statistical test used for **(b-c)** is two-way ANOVA analysis. Statistical differences (*P* values) are indicated in each panel. Source data are provided as a Source Data file.

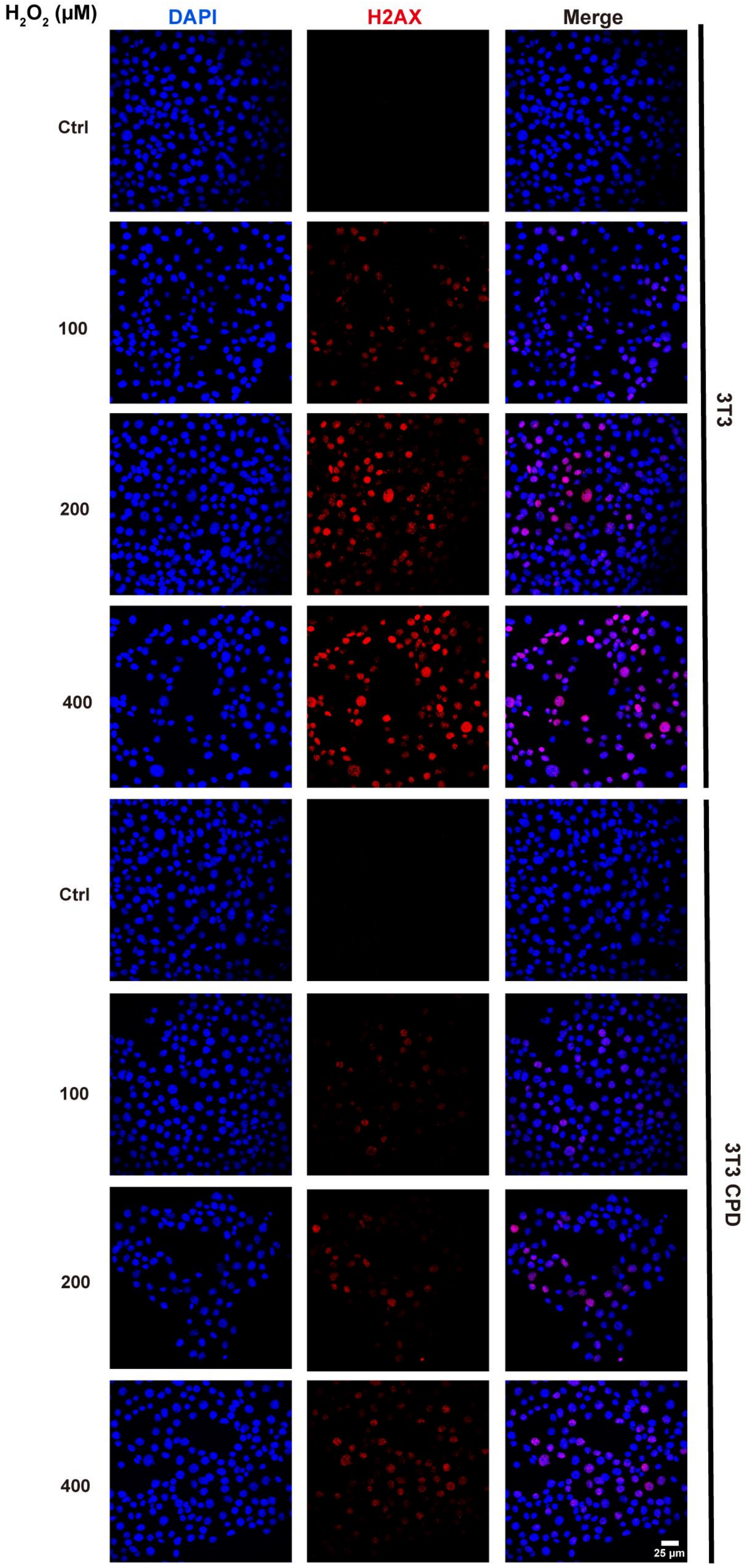

H2O2 (μM)
DAPI
H2AX
Merge
Ctrl
100
200
400
Ctrl
100
200
400
25 μm
3T3
3T3 CPD

66

<u>Supplementary Figure 5</u> Gain of CPDphr function in mammalian cells confers reduced levels of DNA damage upon exposure to oxidative stress. Representative confocal images of 3T3 and 3T3 CPD cell lines immunostained for γ-H2AX (Red) and DAPI (Blue) as well as merged images (Merge) following treatments with various concentrations of H_2O_2 (μM) or in non-treated controls (Ctrl) (as indicated on the left-hand side).

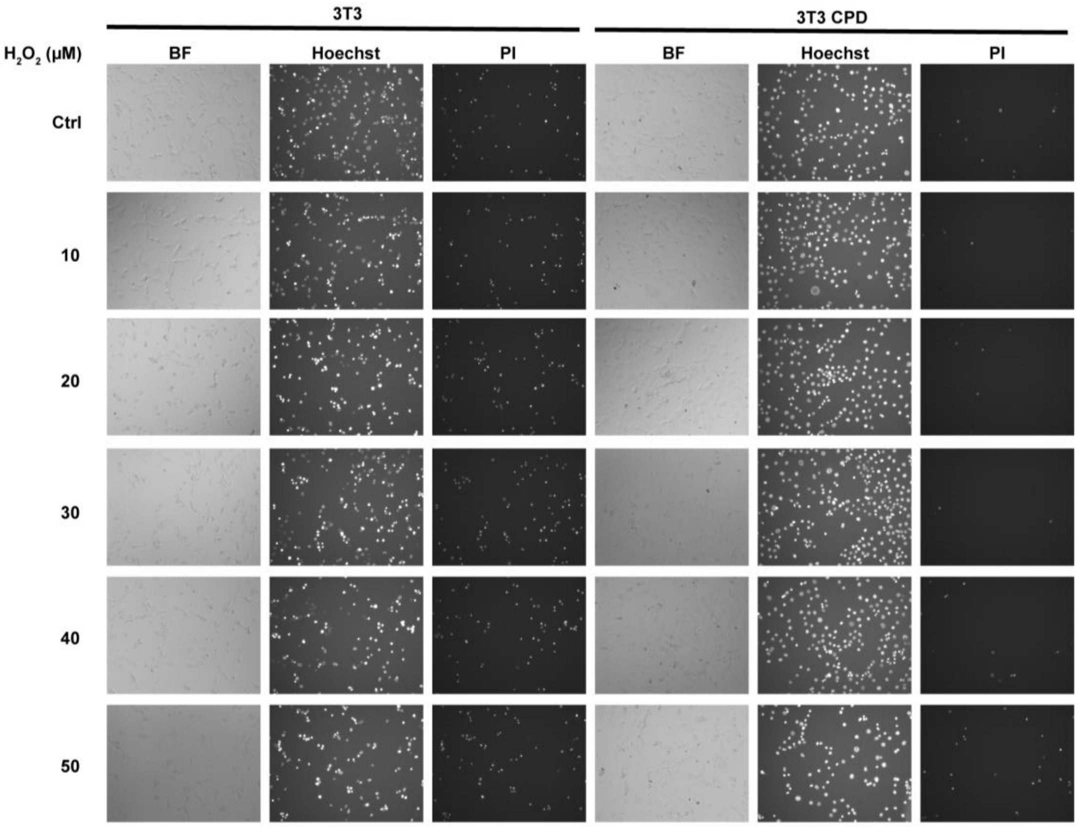

<u>Supplementary Figure 6</u> Gain of CPDphr function in mammalian cells confers enhanced cell survival following exposure to oxidative stress. Representative images from the automated high-throughput microscopy (AHM) assays following exposure of 3T3 and 3T3 CPD cells to different concentrations of H_2O_2 (μM, as indicated on the left-hand side). Bright field (BF) channel, Hoechst staining, and Propidium Iodide (PI) staining are represented.

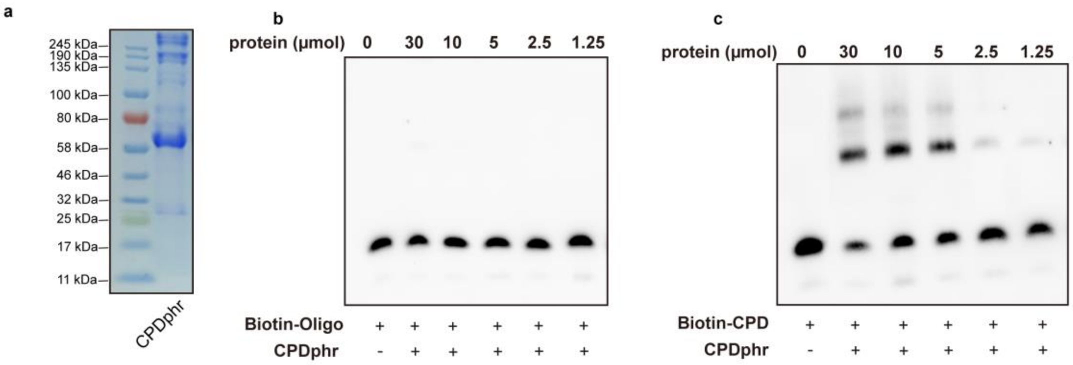

Supplementary Figure 7 Purified zebrafish CPDphr protein has the capacity to bind to CPD photoproduct-containing DNA. (a) Coomassie Blue-stained gel electrophoresis of zebrafish CPDphr protein purified by Ni affinity chromatography following expression in the *E. coli* Er2566 bacterial system. **(b-c)** EMSA (electrophoretic mobility shift assay) of titrated purified zebrafish CPDphr protein (μmol of total protein) with biotin-labelled oligos (nondamaged control oligo (Biotin-Oligo) **(b)** and an oligo carrying CPD photoproducts generated by UV light exposure (Biotin-CPD) **(c)**). All experiments here were repeated at least 3 times, independently and representative images are shown. Source data are provided as a Source Data file.

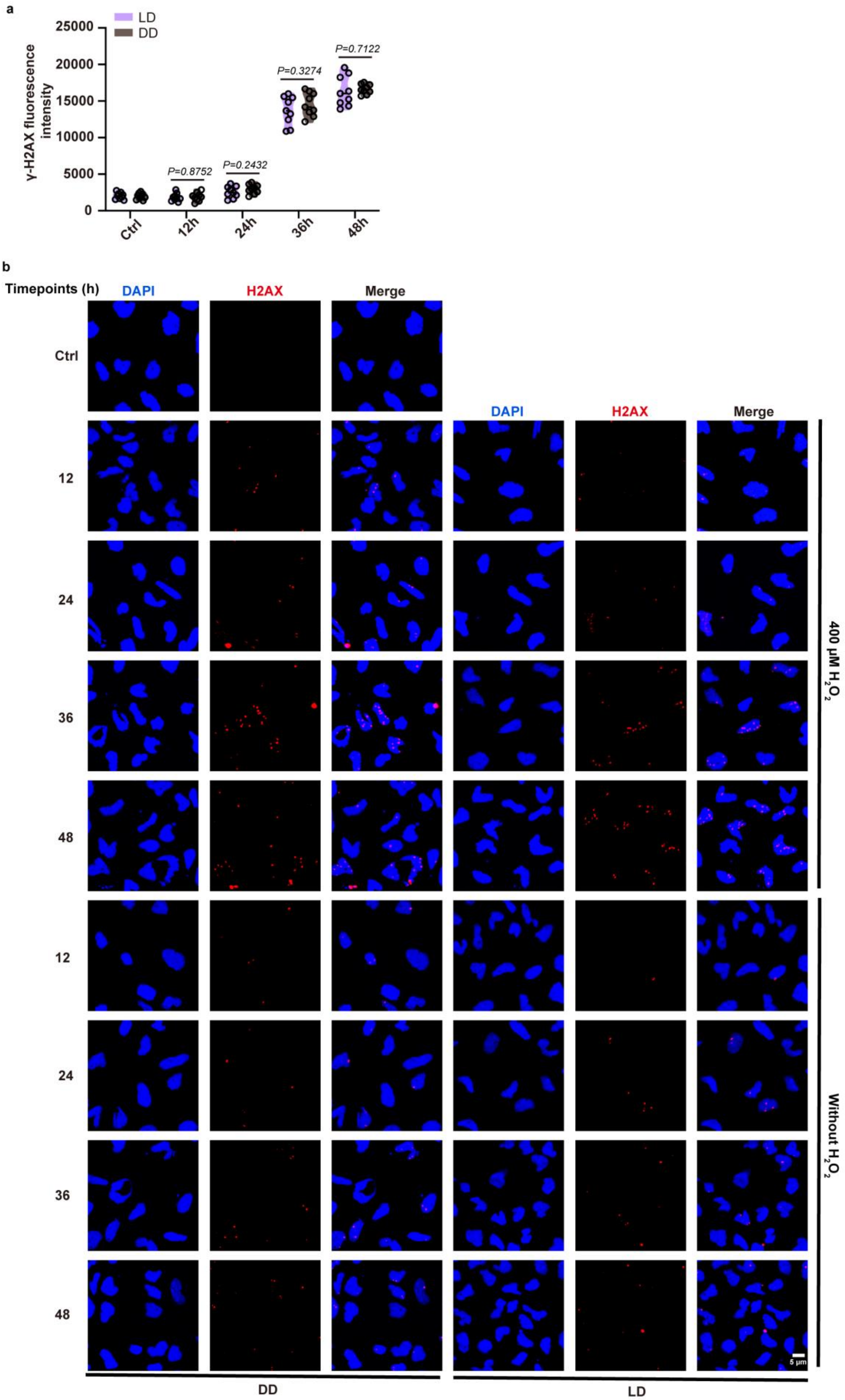
a
LD
DD
γ-H2AX fluorescence intensity
25000
20000
15000
10000
5000
0
P=0.8752
P=0.2432
P=0.3274
P=0.7122
Ctrl
12h
24h
36h
48h
b
Timepoints (h)
DAPI
H2AX
Merge
Ctrl
12
24
36
48
400 μM H2O2
Without H2O2
5 μm
DD
LD

Supplementary Figure 8 **Exposure of medaka cells to light does not influence cell survival following oxidative stress. (a)** Violin plot of immunofluorescence assay of γ-H2AX in medaka WT cells incubated under light-dark cycle (LD) or constant darkness (DD) conditions without H_2O_2 treatment. Cells were fixed at various timepoints as indicated on the x-axis. Mean values of γ-H2AX fluorescence intensity are indicated by black horizontal lines and the black hollow circles represent individual data points (n=9 biologically independent samples). The statistical test used for **(a)** is student's t-test. Statistical differences (*P* values) are indicated in the panel. Source data are provided as a Source Data file. **(b)** Representative confocal images of medaka WT cells exposed transiently to 400 µM H_2O_2 (together with non-treated controls, without H_2O_2) and thereafter left to recover under DD or LD conditions. Nuclear staining (DAPI, Blue), γ-H2AX staining (Red) and the merged images of DAPI and γ-H2AX staining are shown.

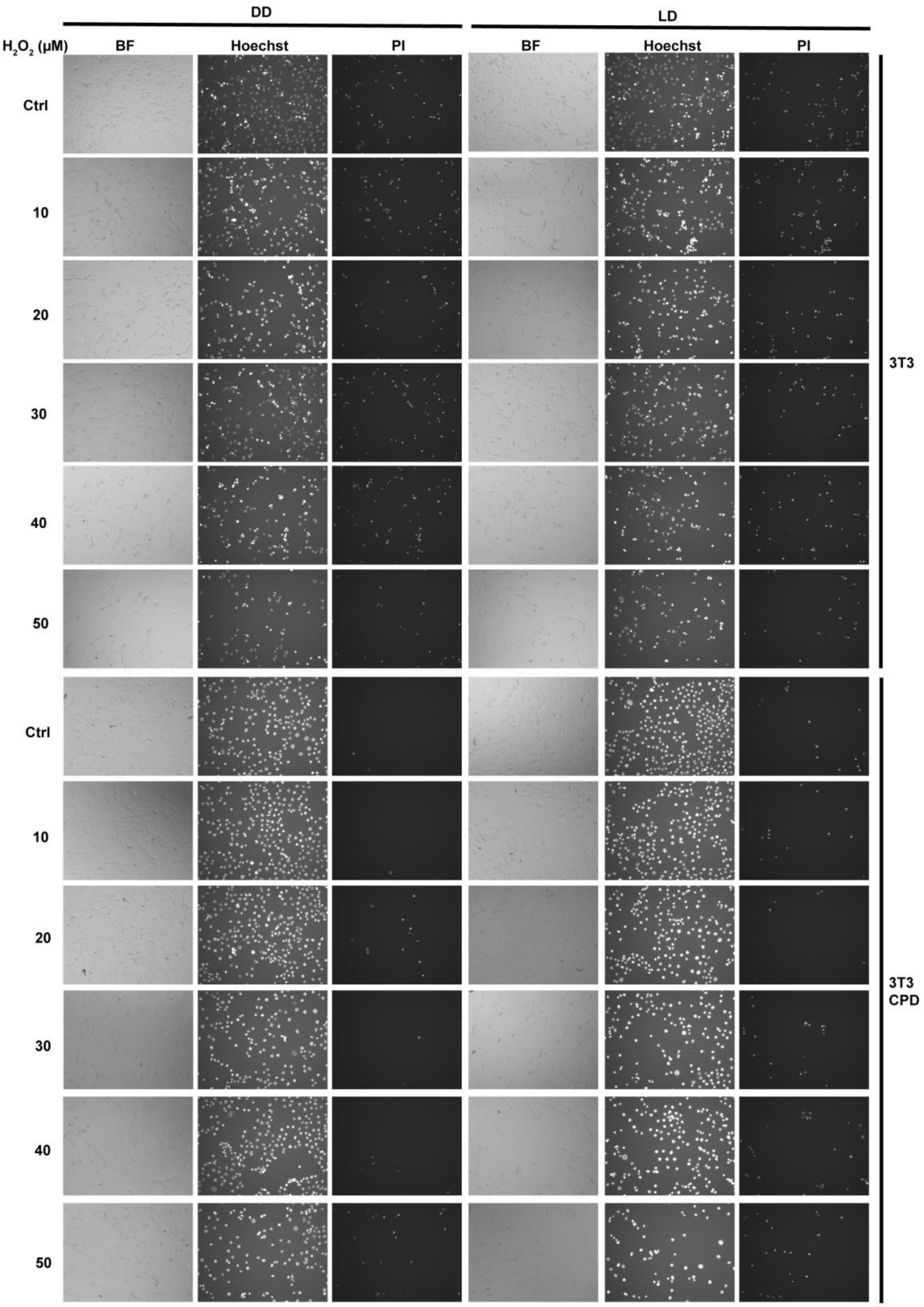

<u>Supplementary Figure 9</u> Light exposure does not influence cell survival following oxidative stress in mammalian cells ectopically expressing CPDphr or in wildtype control cells. Representative images from automated high-throughput microscopy (AHM) assays following exposure of 3T3 and 3T3 CPD cells to different concentrations of H_2O_2 and then recovery under constant

darkness (DD) or light-dark cycle (LD) conditions. Bright field (BF) channel, Hoechst staining, and Propidium Iodide (PI) staining are represented.

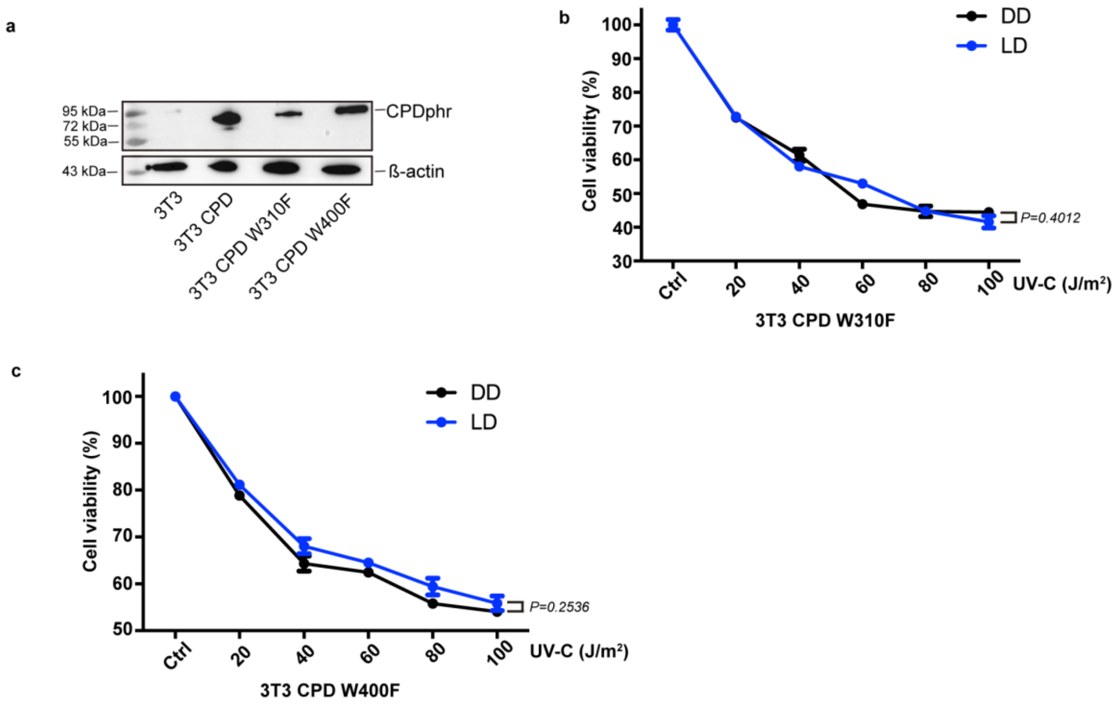

<u>**Supplementary Figure 10**</u> **Ectopic expression of zebrafish CPDphr mutants and the effect of loss of function of the three-tryptophan electron transfer chain upon photoreactivation. (a)** Analysis of the expression of myc-tagged zebrafish CPDphr W310F and W400F mutants in 3T3 cells by western blotting. The expression vectors for the zebrafish CPDphr W310F and W400F mutants were stably transfected into 3T3 cells which were then selected for Neomycin resistance. Non-transfected 3T3 cells were employed as a negative control, while 3T3 CPD cells were used as a positive control. β-actin was used as a loading control. **(b-c)** Cell viability assay of 3T3 cells ectopically expressing CPDphr W310F and W400F mutants revealing loss of photoreactivation function upon UV-C exposure. DD indicates constant darkness, while LD represents light-dark cycle. Mean percentage ± SEM (n=8 biologically independent samples) of cell viability with respect to untreated cells are plotted on the y-axes, while UV-C light doses (from 20 J/m^2 to 100J/m^2) are indicated on the x-axes. All experiments here were repeated at least 3 times, independently and representative data is shown. The statistical test used for

(b-c) is two-way ANOVA analysis. Statistical differences (*P* values) are indicated on each panel. Source data are provided as a Source Data file.

Supplementary tables

Supplementary Table 1 Information of Somalian cavefish *P. andruzzii* colony

seqID	(tank) code	Generation (F)
1	(8A) PH1 2007	2
2	(7B) PH2 2007	2
3	(7C) PH2 2007	2
4	(8B) PH1 2007	2
5	(8C) PH1 2007	2
6	(1B) PH4C1	1
7	(1D) PH4C1	1
8	(2A) NADR16	2
9	(3B) NADR16	2
10	(6A) NADR21	2

All F1 individuals were offspring of wild fish collected in Bud bud (Latitude 04°11’19”N; Longitude 46°28’27”E; at an altitude of 137 m) in 1982. All F2 individuals were offspring of F1 parents which were in turn the progeny of wild fish collected in Bud bud in 1982.

Supplementary Table 2 Primer list for sequence analysis of Somalian cavefish *P. andruzzii*

Name of gene	Primer sequence
CPDphr	F: TCTGCAAACAAACGCAACCTGAA<br>R: TCAACCTGCGCCACATCAAA
6-4phr	F: ATTCACTGGTTTCGCAAGGG<br>R: GGACCCATAGAGGAACACA

<u>Supplementary Table 3</u> Antibody list for immunostaining and western blotting

Antibodies	Company	Catalog number	Dilution
Rabbit phospho-Histone H2A.X (Ser139) monoclonal antibody	Cell Signaling	#9718	1:400
Mouse anti-Myc Tag monoclonal antibody	Merk Millipore	#05-724	1:1000
Mouse anti-β-actin monoclonal antibody	Sigma-Aldrich	#A2228	1:1000
Goat anti-rabbit IgG polyclonal antibody	Jackson ImmunoResearch	#111-001-003	1:200
Horse anti-mouse IgG HRP-linked antibody	Cell Signaling	#7076	1:7500

<u>Supplementary Table 4</u> Primer list for mutagenesis of zebrafish CPDphr

Constructs	Primer sequence
zebrafish CPDphr W310F	F: CCTCTCCCCATTCATTCATGCTGG<br>R: TGGCTAACGGCGTCAGAG
zebrafish CPDphr W400F	F: TGACCAATTGTTTAATGCTGCACAGC<br>R: TGAGTTTCGGCGCTCTCC
