# Supplementary material for: Conservation of dark CPD photolyase function in blind cavefish
Source: Nat Commun. 2025 Aug 11;16:7377. doi: 10.1038/s41467-025-62795-7 (PMC12340131; doi:10.1038/s41467-025-62795-7)
Supplement: Supplementary file 2 — Reporting Summary [file 41467_2025_62795_MOESM2_ESM.pdf]

nature portfolio

Corresponding author(s): Nicholas S. Foulkes

Last updated by author(s): Jul 28, 2025

Reporting Summary

Nature Portfolio wishes to improve the reproducibility of the work that we publish. This form provides structure for consistency and transparency in reporting. For further information on Nature Portfolio policies, see our Editorial Policies and the Editorial Policy Checklist.

Statistics

For all statistical analyses, confirm that the following items are present in the figure legend, table legend, main text, or Methods section.

n/a	Confirmed	
☐	☒	The exact sample size (n) for each experimental group/condition, given as a discrete number and unit of measurement
☒	☐	A statement on whether measurements were taken from distinct samples or whether the same sample was measured repeatedly
☐	☒	The statistical test(s) used AND whether they are one- or two-sided<br>*Only common tests should be described solely by name; describe more complex techniques in the Methods section.*
☒	☐	A description of all covariates tested
☒	☐	A description of any assumptions or corrections, such as tests of normality and adjustment for multiple comparisons
☐	☒	A full description of the statistical parameters including central tendency (e.g. means) or other basic estimates (e.g. regression coefficient) AND variation (e.g. standard deviation) or associated estimates of uncertainty (e.g. confidence intervals)
☐	☒	For null hypothesis testing, the test statistic (e.g. F, t, r) with confidence intervals, effect sizes, degrees of freedom and P value noted<br>*Give P values as exact values whenever suitable.*
☒	☐	For Bayesian analysis, information on the choice of priors and Markov chain Monte Carlo settings
☒	☐	For hierarchical and complex designs, identification of the appropriate level for tests and full reporting of outcomes
☒	☐	Estimates of effect sizes (e.g. Cohen's d, Pearson's r), indicating how they were calculated

Our web collection on statistics for biologists contains articles on many of the points above.

Software and code

Policy information about availability of computer code

Data collection	Immunofluorescence cell and fin clip images were acquired by Leica TCS SP5 confocal microscope and viewed and analyzed by Fiji/Image J software. Cell survival microscopy assay cell images were obtained by automated Olympus IX81 fluorescence microscope and analyzed by scan^R analysis software (version 2.7.3, Olympus, Hamburg, Germany). The absorbance of MTT and ELISA assay was measured by SpectraMax iD3 Microplate Reader. Western blotting and EMSA assay were visualized by ChemiDocTM Imaging System (Bio-Rad). Photolyases libraries were sequenced paired-end (2 x 150 bp) on an Illumina NovaSeq 6000 System.
Data analysis	All data were plotted by using GraphPad Prism 10 (GraphPad Software Inc.) and Excel 2019 and analyzed by SPSS Statistics 19.0 (IBM).

For manuscripts utilizing custom algorithms or software that are central to the research but not yet described in published literature, software must be made available to editors and reviewers. We strongly encourage code deposition in a community repository (e.g. GitHub). See the Nature Portfolio guidelines for submitting code & software for further information.

Data

Policy information about availability of data

All manuscripts must include a data availability statement. This statement should provide the following information, where applicable:

- Accession codes, unique identifiers, or web links for publicly available datasets
- A description of any restrictions on data availability
- For clinical datasets or third party data, please ensure that the statement adheres to our policy

All data acquired or analyzed for this work are included in this published article or provided as source data files. Source data are provided with this paper and the

accession numbers of zebrafish and Somalian cavefish photolyases can be found in figure legend of Figure 1.

Research involving human participants, their data, or biological material

Policy information about studies with human participants or human data. See also policy information about sex, gender (identity/presentation), and sexual orientation and race, ethnicity and racism.

Reporting on sex and gender	N/A
Reporting on race, ethnicity, or other socially relevant groupings	N/A
Population characteristics	N/A
Recruitment	N/A
Ethics oversight	N/A

Note that full information on the approval of the study protocol must also be provided in the manuscript.

Field-specific reporting

Please select the one below that is the best fit for your research. If you are not sure, read the appropriate sections before making your selection.

- [x] Life sciences
- [ ] Behavioural & social sciences
- [ ] Ecological, evolutionary & environmental sciences

For a reference copy of the document with all sections, see nature.com/documents/nr-reporting-summary-flat.pdf

Life sciences study design

All studies must disclose on these points even when the disclosure is negative.

Sample size	IF: 6 or 9. AHM: 4. MTT: 8. ELISA: 6.
Data exclusions	No data were excluded from the analysis.
Replication	All of the experiments were performed by at least 2 or 3 biological repeats and each repeat had at least 3 technical replicates. Data shown in graphs or plots represent mean ± standard error (SE) and plotted data points are shown in violin plots. The statistical significance is considered as the $P < 0.05$. All attempts were successful.
Randomization	The samples were allocated randomly for wild type and gene knock-out mutants.
Blinding	Blinding is not relevant to the experiments in this paper. For all of the experiments, the measurements and analyses were performed identically in all conditions. This paper is mainly for the characterization of evolutionary functionalities of photolyases.

Reporting for specific materials, systems and methods

We require information from authors about some types of materials, experimental systems and methods used in many studies. Here, indicate whether each material, system or method listed is relevant to your study. If you are not sure if a list item applies to your research, read the appropriate section before selecting a response.

Materials & experimental systems

n/a	Involved in the study	
	x	Antibodies
	x	Eukaryotic cell lines
x		Palaeontology and archaeology
	x	Animals and other organisms
x		Clinical data
x		Dual use research of concern
x		Plants

Methods

n/a	Involved in the study	
x		ChIP-seq
x		Flow cytometry
x		MRI-based neuroimaging

Antibodies

Antibodies used	Rabbit phospho-Histone H2A.X (Ser139) monoclonal antibody (#9718, Cell Signaling)<br>Mouse anti-Myc Tag monoclonal antibody, clone 4A6 (#05-724, Merck Millipore)

	Mouse anti-β-actin monoclonal antibody (#A2228, Sigma-Aldrich)<br>Goat anti-rabbit IgG polyclonal antibody (#111-001-003, Jackson ImmunoResearch)<br>Horse anti-mouse IgG HRP-linked antibody (#7076, Cell Signaling)
Validation	These antibodies are commercially available and have been extensively validated. The rabbit phospho-Histone H2A.X (Ser139) monoclonal antibody is validated for WB, IHC, IF and F in species including human, mouse, rat and monkey (https://www.cellsignal.com/products/primary-antibodies/phospho-histone-h2a-x-ser139-20e3-rabbit-mab/9718?srsltid=AfmBOoqv3ehWmswaCCYEjNOoqm2fW8hK8J30w8Is2w9fBOgVFIU9eeSJ). The mouse anti-Myc Tag monoclonal antibody, clone 4A6, is validated for use in ChIP, IC, IF, IP and WB for the detection of Myc Tag and does not show the very high context sensitivity of 9E10 (https://www.merckmillipore.com/DE/de/product/Anti-Myc-Tag-Antibody-clone-4A6,MM_NF-05-724?ReferrerURL=https%3A%2F%2Fwww.google.com.hk%2F). The mouse anti-β-actin monoclonal antibody is validated in species including rabbit, sheep, cat, guinea pig, bovine, mouse, chicken, wide range, Hirudo medicinalis, canine, Drosophila, carp, pig, rat, human (https://www.sigmaaldrich.com/DE/de/product/sigma/a2228?srsltid=AfmBOoqoErS5OaROT5ctGmHXZxnfGwok4mmAN8MrxCQ789KGZ1MXF5qW).

Eukaryotic cell lines

Policy information about cell lines and Sex and Gender in Research

Cell line source(s)	CPD WT is derived from embryos of medaka CPD WT fish<br>CPD KO is derived from embryos of medaka CPD KO fish<br>DASH WT is derived from embryos of medaka DASH WT fish<br>DASH KO is derived from embryos of medaka DASH KO fish<br>6-4 WT is derived from embryos of medaka 6-4 WT fish<br>6-4 KO is derived from embryos of medaka 6-4 KO fish<br>3T3 originally came from the ATCC Cell Biology collection<br>3T3 CPD is derived from 3T3 cells transfected with zebrafish CPDphr and selected by neomycin<br>3T3 CPD W310F is derived from 3T3 cells transfected with zebrafish CPDphr W310F mutation and selected by neomycin<br>3T3 CPD W400F is derived from 3T3 cells transfected with zebrafish CPDphr W400F mutation and selected by neomycin<br>The sex of all cell lines is N/A
Authentication	All medaka cell lines were authenticated by the publication below (https://onlinelibrary.wiley.com/doi/10.1111/php.12658)<br>The functional authentication of 3T3 CPD, 3T3 CPD W310F and 3T3 CPD W400F cell lines is provided in this paper
Mycoplasma contamination	All cell lines tested negative for mycoplasma contamination.
Commonly misidentified lines<br>(See ICLAC register)	N/A

Animals and other research organisms

Policy information about studies involving animals; ARRIVE guidelines recommended for reporting animal research, and Sex and Gender in Research

Laboratory animals	zebrafish (D. rerio), Somalian cavefish (P. andruzzii), medaka (O. latipes) iCab, medaka CPD KO, medaka DASH KO and medaka 6-4 KO
Wild animals	The study did not involve wild animals.
Reporting on sex	The sex in zebrafish, Somalian cavefish and medaka was not considered.
Field-collected samples	The study did not involve samples collected from the field.
Ethics oversight	The experiments performed with fish were conducted in compliance with the European Legislation for the Protection of Animals used for Scientific Purposes (Directive 2010/63/EU) (General license for fish maintenance and breeding: Az.: 35-9185.64/ BH KIT IBCS-BIP) and adhered to the animal protection standards of Germany. The permission to perform experiments with these medaka mutant lines was approved under the license: 35-9185.81/G-132/21 and 35-9185.81/G-149/23 KIT IBCS-BIP.<br>The permission to perform experiments with cavefish was approved by the Italian Ministry of Health (aut. N. 890/2016-PR) and were conducted in compliance with European (Directive 2010/63/EU) and Italian legislation.

Note that full information on the approval of the study protocol must also be provided in the manuscript.

Plants

Seed stocks	N/A
Novel plant genotypes	N/A
Authentication	N/A
